# Astaxanthin Improves Human Sperm Capacitation by Inducing Lyn Displacement and Activation

**DOI:** 10.3390/md13095533

**Published:** 2015-08-25

**Authors:** Alessandra Andrisani, Gabriella Donà, Elena Tibaldi, Anna Maria Brunati, Chiara Sabbadin, Decio Armanini, Gualtiero Alvisi, Salvatore Gizzo, Guido Ambrosini, Eugenio Ragazzi, Luciana Bordin

**Affiliations:** 1 Department of Women’s and Chilren’s Health, University of Padova, Padova 35100, Italy; E-Mails: alessandra.andrisani@unipd.it (A.A.); salvatore.gizzo@gmail.com (S.G.); guido.ambrosini@unipd.it (G.A.); 2 Department of Molecular Medicine-Biological Chemistry, University of Padova, Padova 35129, Italy; E-Mails: gabriella.dona@unipd.it (G.D); elena.tibaldi@unipd.it (E.T); annamaria.brunati@unipd.it (A.M.B.); 3 Department of Medicine-Endocrinology, University of Padova, Padova 35100, Italy; E-Mails: ChiaraSabbadin@libero.it (C.S.); decio.armanini@unipd.it (D.A.); 4 Department of Molecular Medicine-Microbiology Section, University of Padova, Padova 35129, Italy; E-Mail: gualtiero.alvisi@unipd.it; 5 Department of Pharmaceutical and Pharmacological Sciences, University of Padova, Padova 35129, Italy; E-Mail: eugenio.ragazzi@unipd.it

**Keywords:** astaxanthin, tyrosine kinase Lyn, human sperm capacitation, acrosome reaction, cholera toxin subunit B (CTB)

## Abstract

Astaxanthin (Asta), a photo-protective red pigment of the carotenoid family, is known for its multiple beneficial properties. In this study, the effects of Asta on isolated human sperm were evaluated. Capacitation involves a series of transformations to let sperm acquire the correct features for potential oocyte fertilization, including the generation of a controlled amount of reactive oxygen species (ROS), cholesterol depletion of the sperm outer membrane, and protein tyrosine phosphorylation (Tyr-P) process in the head region. Volunteers, with normal spermiogram values, were divided in two separate groups on the basis of their ability to generate the correct content of endogenous ROS. Both patient group (PG) and control group (CG) were analysed for Tyr-phosphorylation (Tyr-P) pattern and percentages of acrosome-reacted cells (ARC) and non-viable cells (NVC), in the presence or absence of Asta. In addition, the involvement of ROS on membrane reorganization and the presence of Lyn, a Src family kinase associated with lipid rafts, were investigated. Results show that Lyn is present in the membranes of human sperm, mainly confined in midpiece in resting conditions. Following capacitation, Lyn translocated to the head concomitantly with raft relocation, thus allowing the Tyr-P of head proteins. Asta succeeded to trigger Lyn translocation in PG sperm thus bypassing the impaired ROS-related mechanism for rafts and Lyn translocation. In this study, we showed an interdependence between ROS generation and lipid rafts and Lyn relocation leading the cells to undergo the successive acrosome reaction (AR). Asta, by ameliorating PG sperm functioning, may be utilised to decrease male idiopathic infertility.

## 1. Introduction

Routinely performed sperm tests, such as semen volume, colour, pH, liquefaction time, viscosity, sperm count and motility, sperm morphology, concentration of round cells and polymorphonucleocytes, sperm agglutination and sperm viability, often do not explain male inability to achieve a pregnancy after 12 months or more of regular, unprotected and well-timed intercourse. The term idiopathic infertility is due to the fact that results of these tests typically fall within the normal range without clarifying the exact reason why patients result infertile/subfertile [1].

In order to acquire the correct features for potential oocyte fertilization, human sperm has to undergo a series of transformations, known as capacitation, which prepares the cell to undergo the acrosome reaction (AR). During the capacitation process, the sperm outer membrane undergoes cholesterol depletion [2,3], to augment membrane fusibility and enhance the acrosomal exocytosis, followed by downstream signaling processes such as protein serine and tyrosine phosphorylation (Tyr-P) [4,5,6] and reactive oxygen species (ROS) generation [7].

Capacitation-related efflux of cholesterol, a major component of lipid rafts [8,9], might also change membrane fluidity and/or induce rearrangement of membrane lipid rafts [10,11].

Generally, lipid rafts are small microdomains ranging from 10 to 200 nm in size characterized by the ganglioside GM1, a raft constituent and marker, which, by binding to the fluorescence-conjugated cholera toxin B subunit (CTB) [12], may be identified and monitored during capacitation transformations [13].

Caveolin 1 (CAV 1) is one of rafts’ constituents [14] and provides the scaffolding of these microdomains, also called caveolae [15], which can embed and inactivate many proteins and enzymes [14].

High ROS level and oxidative stress have been implicated in the pathophysiology of male infertility [16] being correlated with sperm DNA damage, reduced sperm motility, and lipid and protein denaturation [16,17]. However, growing evidence has been recently proposed [18] about the essential role of a controlled ROS generation on the correct sperm functioning, thus giving ROS a key role in the maturation process, other than the detrimental factor previously assessed [19].

Moreover, the time-dependent ROS generation has been demonstrated to represent an important tool to predict the potential ability of the cell to accomplish acrosome reaction [13,18,19,20], discriminating sperms that, either for lower or higher ROS content, were prevented from being capacitated [18]. In addition, the close relationship between the Tyr-P of the sperm head and AR has been demonstrated [18,19], although the molecular mechanisms involved in all these capacitation-related processes remain to be characterized. 

Phosphorylation is among the most common regulatory mechanisms for protein function, regulating cell functions by inducing conformational changes in proteins via allosteric modification [21]. In human sperm, several members of the Src kinase family have recently been described [22,23,24], but, despite the importance of the events triggered by Tyr-P such as hyperactivation [24], capacitation progress and acrosome reaction regulation [25], protein tyrosine kinases (PTKs) identification remains to be completely elucidated. At present, the most well characterized mechanisms of Tyr-P are regulated by members of the Src family kinases (SFK), which is the largest family of non-receptor tyrosine kinases widely expressed in many cell types and in different subcellular compartments [26]. SFKs induce cellular responses associated with proliferation, growth control, survival, differentiation and cytoskeletal arrangements [26] and many of their own functions have been clarified with the use of inhibitors of their activities, such as 4-Amino-5-(4-chlorophenyl)-7-(*t*-butyl)pyrazolo[3,4-d]pyrimidine (PP2) [27].

While the role of hormone therapy for men with an identified abnormality is well defined [28], the literature remains inconclusive and controversial regarding the use of food supplements for men with idiopathic infertility, mainly due to the incomplete knowledge of all the steps of the capacitation process.

Many medical therapies have been historically used for male infertility, including herbs, vitamins, and nutritional supplements, and many of them rely on antioxidant properties. Astaxanthin (Asta), a carotenoid nutrient widely distributed in algae, crustaceans, shellfish and various plants [29], inserts within cell membranes protecting them with potent antioxidant and anti-inflammatory actions [30,31]. In a recent study [20], we described that Asta ameliorates viability and AR percentages of human sperm. To test the hypothesis of whether Asta could induce protein-protein or protein-lipid dislocation at the plasma membrane level, in the present study we evaluated the effect of Asta on sperm membrane raft reorganization, in particular at the level of head/acrosome region. In addition, the presence of Lyn, a Src family kinase found in lipid rafts [32,33], was investigated as potential Tyr-kinase involved in the capacitation-related Tyr-P.

## 2. Results and Discussion

We have previously described that Asta can improve human sperm viability in a dose-dependent manner with 2 μM dose representing optimal concentration for capacitation [20].

After gradient separation, sperm samples of each group were collected three by three in a single pool to obtain a sufficient number of cells to perform all tests. Pools were divided in samples which were immediately analysed (T_0_), or incubated in the absence (C), or presence of Asta, PP2, an inhibitor of the Src family kinase, or both (Asta + PP2). Before any further analysis, aliquots were then assessed for sperm parameters by computer-assisted sperm analysis (CASA) (Supplementary Table S1). As expected, samples C showed a net increase in the different motility parameters compared with T_0_, thus confirming that capacitation-related hyper-activation was occurred. No significant alteration was evident by comparing capacitated sperm (C) with those with Asta, or Asta + PP2, except for a minor increase in patient group (PG) sperm treated with Asta, thus suggesting that hyperactivity was almost not affected by these different conditions.

Another aliquot of each sample was analysed for the ROS generation in a luminometer for 180 min with luminol as a luminescent source. Samples C generating ROS in a concentration sufficient to induced luminescence between the values 0.05–0.08 Relative Luminescence Units (RLU) were considered normal [18] and, therefore, belonging to the control group (CG, *n* = 15), whereas samples expressing ROS below these values were inserted in the patient group (PG, *n* = 18). On the basis of this classification, samples which did not meet the above criteria were discarded.

**Figure 1:**
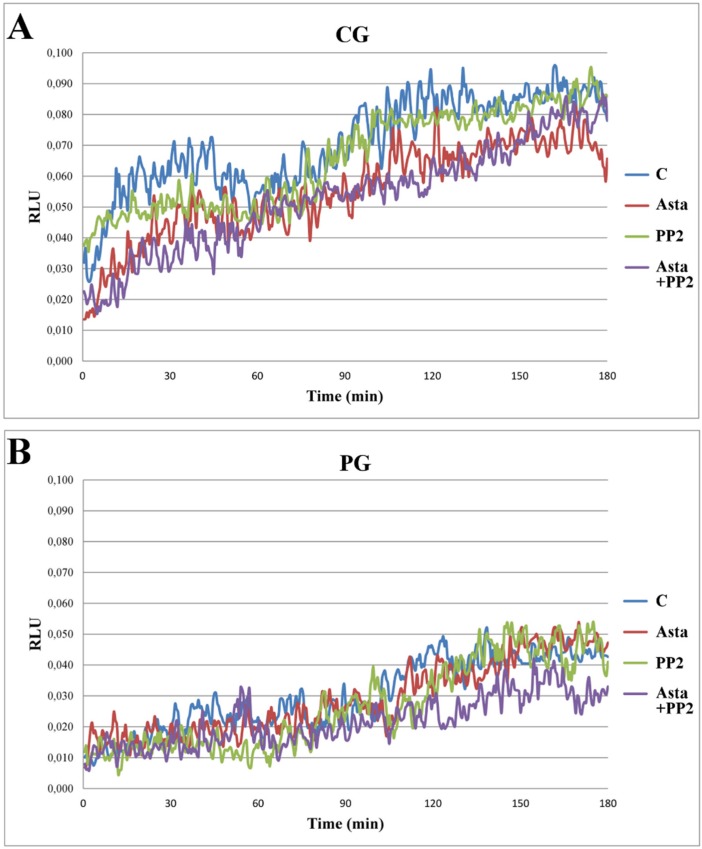

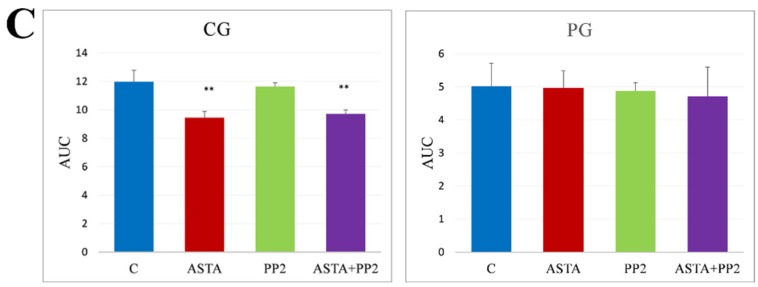
ROS generation curves of sperm samples and effects of Asta, PP2, or both. Sperm from three volunteers for each experiment was collected to form a pool with a sufficient number of cells. Sperm was incubated for up to 180 min in capacitating conditions in the absence (C) or presence of Asta (2 μM), PP2 (6 μM), or both (Asta + PP2, 2 μM + 6 μM). Luminol chemiluminescence was monitored during sperm capacitation. Results are expressed as moving averages of Relative Luminescence Units (RLU)/30 s for 2 × 10^6^ cells. Figure 1 is representative of 11 separate experiments, conducted on samples belonging to the control group (CG, panel **A**) or the patient group (PG, panel **B**). Detection was carried out in triplicate. Graphs in panel **C** show AUC of the RLU data in both groups. ** *p* < 0.01 *vs.* C; Student’s *t* test.

When analysed in the presence of Asta, no relevant alteration in the curves of ROS generation (Figure 1) was observed in either CG (panel A) or PG (panel B) groups, although evaluating the integrated values (as area under the curve, AUC) a modest, but significant (*p* < 0.01), reduction was found in RLU of CG group, also in the presence of both Asta + PP2 (panel C). This fact suggests that Asta has a modest role in preventing H_2_O_2_ formation in sperm in physiological condition but no effect in patients.

The close relation between the endogenous ROS content (Supplementary Figure S1) and sperm viability (Supplementary Table S2) were further investigated by incubating CG sperm in the presence of ascorbic acid (AA), and PG sperm with H_2_O_2_ (0.25 μM). As previously reported [18], AA greatly reduced ROS content (panel A) and prevented capacitation as shown by Tyr-P and AR (Supplementary Table S2). On the other hand, when PG sperm was treated in the presence of H_2_O_2_ to ameliorate ROS content (panel B) and trigger capacitation, although a peak within the region of correct values of ROS was formed, no improvement either in Tyr-P level and distribution or acrosome reacted cells (ARC) was shown (Supplementary Table S2). In this case, it is hypothesized that the impairment, present in PG sperm, was not accessible by exogenous ROS addition, probably due to its inner insertion within the membrane. Higher ROS doses were not consistent with cell survival [18] as suggested by the increase of non-viable cells (NVC) percentage (Supplementary Table S2).

At the end of incubation, aliquots of cells were withdrawn from all samples and analyzed for their ability to undergo Tyr-P and AR.

The Tyr-P pattern was evaluated (Supplementary Figure S2 and Table S1). According to previous reports [19] (Supplementary Figure S2, panel A), capacitation induced a net increase in the total cell lysate Tyr-P level of two main bands at about 90 and 110 kDa, respectively, in the CG, with Asta showing only a slight further enhancement and PP2 completely preventing any increase either alone or in co-addition with Asta (lanes PP2 and Asta + PP2, respectively). On the other hand, in the PG, capacitation-related increase of the Tyr-P was clearly lower (only 20% compared with T_0_, *p* < 0.001) with Asta inducing a net increase (40% compared with T_0_, *p* < 0.001). PP2 completely inhibited Tyr-P increase in any conditions (lanes PP2 and Asta + PP2).

When cells were analysed for Tyr-P distribution with immunocytochemistry (Supplementary Figure S2), as previously reported [19], in sample T_0_, Tyr-P was mainly located in the mid-piece region and tails of spermatozoa [19] and involved about 95% of the cells (Table 1, Tyr-P cells) in CG but only 48% of cells in PG. Cells presenting Tyr-P also in the region of head (Tyr-P head) were about 10% or less in both CG and PG. After 180 min in capacitating conditions (C), only a few cells of PG presented Tyr-P in the head region (19% ± 3%), as expected on the basis of ROS generation values expressed by cells (Figure 1B), compared to C sample of CG (63% ± 6%). Interestingly, the addition of Asta clearly increased the percentage of PG sperm presenting Tyr-P in head (27% ± 3% compared to 19% ± 3% in C, *p* < 0.0001) although ROS generation content was not affected at all (Figure 1B). On the contrary, in CG samples, Asta did not induce any significant increase compared with C condition. When added, PP2, either alone or in co-addition with Asta, drastically diminished the percentage of cells presenting Tyr-P in both groups (Table 1), thus confirming the involvement of Src family kinase in the capacitation-related Tyr-P process.

**Table 1:** Sperm cells from control group (CG) or patient group (PG) at T_0,_ or incubated for 180 min in capacitating conditions in the absence (C) or presence of Asta 2 μM, PP2 6 μM or both (Asta + PP2), were analyzed for Tyr-P pattern (Supplementary Figure S2), acrosome-reacted cells (ARC) and viability (non-viable cells, NVC) by immunofluorescence cytochemistry (see Methods). Number of cells expressed as % of total number of cells showing Tyr-P in any part of cell body or in head, were detected and reported as Tyr-P cells and Tyr-P head, respectively. Percentages of cells undergoing acrosome reaction (ARC) or NVC were also reported. Values are expressed as means ± SD. ^†^ *p* < 0.01; ^††^ *p* < 0.001; ^‡^ *p* < 0.0001 comparison between various samples *vs.* C as reference; Student’s *t*-test for paired data. * *p* < 0.05; ** *p* < 0.001; *** *p* < 0.0001 comparison C *vs.* T_0_; Student’s *t*-test for paired data.

Groups	Parameters	T0	C	Asta	PP2	Asta + PP2
**CG**	**Tyr-P cells**	95 ± 3	93 ± 4	94 ± 5	30 ± 6 ^††^	36 ± 7 ^‡^
	**Tyr-P head**	10 ± 2	63 ± 6 ***	67 ± 5	4 ± 2 ^‡^	5 ± 2 ^‡^
	**ARC**	16 ± 5	59 ± 7 ***	63 ± 6	5 ± 2 ^‡^	6 ± 1 ^‡^
	**NVC**	7 ± 5	9 ± 3	5 ± 2 ^†^	69 ± 5 ^‡^	57 ± 7 ^††^
**PG**	**Tyr-P cells**	48 ± 6	57 ± 5	64 ± 5	24 ± 6 ^‡^	29 ± 5 ^‡^
	**Tyr-P head**	8 ± 2	19 ± 3 **	27 ± 3 ^‡^	2 ± 1 ^‡^	3 ± 1 ^††^
	**ARC**	10 ± 4	15 ± 4 **	31 ± 4 ^‡^	4 ± 2 ^††^	4 ± 1 ^††^
	**NVC**	13 ± 4	19 ± 2 *	12 ± 2 ^††^	65 ± 8 ^‡^	58 ± 4 ^‡^

Aliquots of sperm suspensions were analyzed for percentages of ARC and NVC. The values of cell counts are reported in Table 1. As previously reported [19], the percentages of cells which underwent acrosome reaction (ARC) had a trend similar to the corresponding values of Tyr-P in acrosome/head region, once more confirming the correlation existing between these two parameters. Asta, ameliorating the percentage of Tyr-P cells in PG, increased ARC values in this group (31% ± 4% *vs.* 15% ± 4%, *p* < 0.0001) but in CG, Asta did not induce any increase, compared to C (63% ± 6% *vs.* 59% ± 7%, respectively). PP2, both alone or in co-addition with Asta, prevented cells from AR, as indicated by the low percentages of ARC (5% ± 2% or 6% ± 1% compared to 59% ± 7% of the C sample in CG, *p* < 0.0001; 4% ± 2% or 4% ± 1% compared to 15% ± 4% of the C sample in PG, *p* < 0.001) and seriously compromised cell viability, as shown by the high number of NVC (69% ± 5% compared to 9% ± 3% of the C sample in CG, *p* < 0.0001; 65% ± 8% compared to 19% ± 2% of the C sample in PG, *p* < 0.001) also in the presence of Asta. Results showed that Asta was an optimal support for PG sperm to achieve capacitation status even in the absence of the correct ROS amount. Addition of Asta increased both Tyr-P and ARC percentages with significant increase of PG-C values (Tyr-P head 27% ± 3% in the presence of Asta, compared to 19% ± 3% of the corresponding sample C, and ARC 31% ± 4% compared to 15% ± 4%, in the presence or absence of Asta, respectively). When added, PP2 greatly augmented the percentages of NVC in both groups, which also showed a net decrease in all the other parameters. Asta did not succeed in reversing PP2 negative effects on sperm functioning and viability.

**Figure 2:**
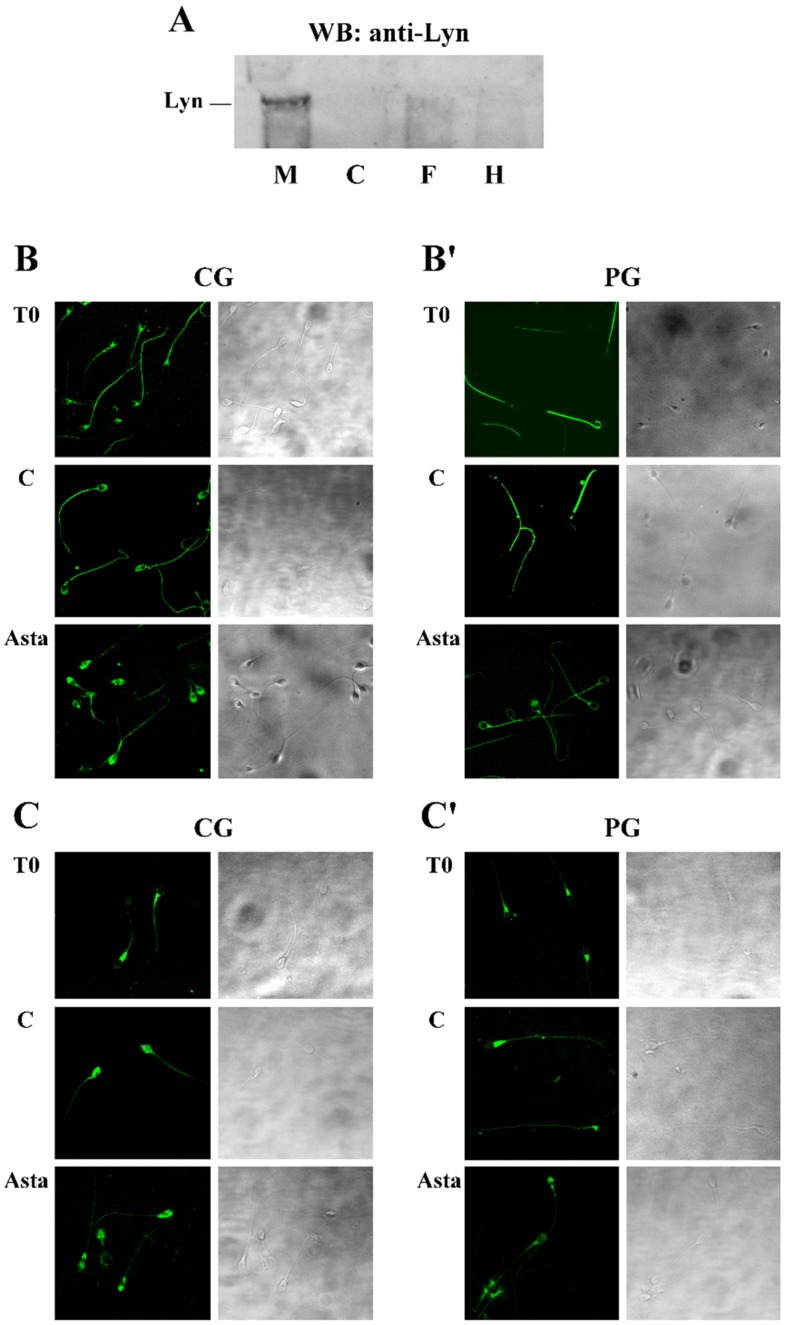
Subcellular localization of Lyn (panel **A**) and membrane rafts (panels **B** and **B’**) and Lyn (panels **C** and **C’**) translocation in human sperm during capacitation in absence or presence of Asta. (Panel **A**): Western blot analysis of subcellular localization of human sperm Lyn. The presence of the Src family kinase Lyn was assessed in the non-capacitated sperm plasma membrane (M), cytosol (C), flagellum (F) or head (H), obtained as described in Methods. Aliquots from each fraction corresponding to 3 × 10^6^ cells were loaded and analysed by SDS-PAGE (10%), transferred to nitrocellulose and immuno-revealed with anti-Lyn antibody. (Panels **B, B’, C,** and **C’**): Sperm cells from control group (CG, panels **B** and **C**) and patient group (PG, panels **B’** and **C’**), at T_0_ or incubated in capacitating conditions for 180 min in absence (C) or presence of Asta (2 μM), were analysed for CTB labelling (panels **B** and **B’**) and Lyn localization (panels **C** and **C’**) by immunofluorescence cytochemistry as described in Methods. Corresponding phase-contrast images for each condition are shown. The figure is representative of 11 separate experiments conducted in triplicate.

Taking into account that, in other cells, rafts contain Lyn, another member of the Src family kinase, we investigated the presence and the potential involvement of this enzyme in sperm functioning. In order to determine if the enzyme was expressed all over sperm surface or in precise cellular compartments, sperm was separated in membranes (M), cytosol (C), head (H) and flagellum (F), and successively analysed by anti-Lyn antibody in a Western blotting assay. As indicated by Figure 2 panel A, Lyn was present in human sperm with a subcellular localization in the plasma M, enveloping the cell. For this reason, to further inquire about any potential alteration of Lyn position within the cells following treatments, differently treated sperm samples were analysed with anti-Lyn and visualized by confocal microscopy, to localize the enzyme (Figure 2). Sperm was also inquired with CTB, which, by binding to membrane-inserted protein G1, allowed researchers to localize rafts and capacitation-related raft translocation [13].

At T_0_, Lyn was predominantly located at the neck of the sperm in both CG and PG, but after 180 min of capacitation, Lyn spread over sperm heads, locating predominantly to the acrosome region in CG, whereas, in PG, it remained in the neck or, at most, in the pre-acrosomal region, only rarely reaching the acrosome region (Figure 2 and Table 2 for quantification). Asta increased the percentage of cells from PG presenting both CTB and Lyn in the acrosome region, from 12% ± 2% to 28% ± 7% (CTB) and from 15% ± 2% to 22% ± 1% (Lyn) of samples C and Asta, respectively. These percentages accounted for what observed in PG Tyr-P and ARC (Table 1). Interestingly, PP2, the inhibitor of Src kinase activity, did not cause any Lyn displacement alteration either in the absence or presence of Asta in PG and CG, thus confirming that Lyn activity was not a prerequisite for its translocation. However, even if Lyn spread over the acrosome region, a net inhibition of acrosome reaction was observed, as indicated by the percentage of ARC (5% ± 2% in PP2 and 6% ± 1% in Asta + PP2), compared to capacitated (C) samples (59% ± 7%).

The relative tyrosine kinase activities in samples from differently treated cells were also evaluated (Table 2). Cells were lysed, and lysates were assayed as Protein Tyrosine Kinase (PTK) activity in the total cell lysate, or, after immuno-precipitation (Ip) with anti-Lyn antibodies (Lyn-Ip). Results showed that capacitating conditions (C) induced a net increase of both total lysate and Lyn activities in CG (+73% and +81%, respectively, compared to T_0_ conditions), by far higher than those evidenced in PG (+24% and +21%, compared to T_0_) (Table 2). Asta addition induced further increase of both total lysate and Lyn activities in PG (+16% in both samples, compared to C, *p* < 0.01), but not in CG.

**Table 2:** Membrane rafts localization, Lyn localization and activity in human sperm during capacitation and effects of Asta, PP2, or both. Sperm cells from control group (CG, panel **A**) and patient group (PG, panel **B**), at T_0_ or incubated in capacitating conditions for 180 min in absence (C) or presence of Asta 2 μM (Asta), PP2 6 μM (PP2) or both (Asta + PP2), were analysed for cholera toxin subunit B (CTB) labelling and Lyn localization by immunofluorescence cytochemistry as described in Methods. Number of cells, expressed as % of the total amount of cells showing labelling in acrosome, sub-acrosomal region (Sub-acro), neck and tail, were detected and reported. Protein Tyrosine Kinase (PTK) activity: aliquots of total sperm lysate or Lyn-Ip from each sample were analyzed for PTK activity as described in Methods. The values were obtained as the ratio percentage of PTK activity of samples to T_0_ (chosen as arbitrary comparison units). The figure is representative of 11 separate experiments. Values are expressed as means ± SD. ^†^ *p* < 0.05; ^††^ *p* < 0.01; ^‡^ *p* < 0.001 comparison between various samples *vs.* C as reference; Student’s *t*-test for paired data. * *p* < 0.05; ** *p* < 0.01; *** *p* < 0.001 comparison C *vs.* T_0_; Student’s *t*-test for paired data.

**A**		**Localization**			**PTK Activity**	
	**Treatment**	**Compartment**	**CTB (%)**	**Lyn (%)**	**Total Lysate**	**Lyn-Ip**
**CG**	**T0**	**Acrosome**	0 ± 0	0 ± 0	100 ± 1	101 ± 2
		**Sub-acro**	1 ± 1	4 ± 2		
		**Neck**	89 ± 8	96 ± 4		
		**Tail**	92 ± 4	11 ± 6		
	**C**	**Acrosome**	91 ± 8 ***	93 ± 6 ***	173 ± 9 ***	181 ± 7 ***
		**Sub-acro**	91 ± 5 ***	90 ± 8 ***		
		**Neck**	92 ± 4	77 ± 6 ***		
		**Tail**	78 ± 10	9 ± 4		
	**Asta**	**Acrosome**	95 ± 3	91 ± 5	178 ± 11	183 ± 8
		**Sub-acro**	92 ± 2	93 ± 6		
		**Neck**	90 ± 6	72 ± 7		
		**Tail**	76 ± 7	10 ± 5		
	**PP2**	**Acrosome**	31 ± 8 ^‡^	26 ± 8 ^‡^	29 ± 6 ^‡^	2 ± 1 ^‡^
		**Sub-acro**	37 ± 11 ^††^	29 ± 3 ^‡^		
		**Neck**	79 ± 10 ^†^	89 ± 7		
		**Tail**	78 ± 9	7 ± 3		
	**Asta + PP2**	**Acrosome**	52 ± 7 ^‡^	34 ± 5 ^‡^	30 ± 5 ^‡^	1 ± 1 ^‡^
		**Sub-acro**	54 ± 6 ^‡^	31 ± 6 ^‡^		
		**Neck**	85 ± 9	79 ± 5		
		**Tail**	84 ± 5	8 ± 4		
**B**		**Localization**			**PTK Activity**	
	**Treatment**	**Compartment**	**CTB (%)**	**Lyn (%)**	**Total Lysate**	**Lyn-Ip**
**PG**	**T0**	**Acrosome**	0 ± 0	0 ± 0	101 ± 1	102 ± 1
		**Sub-acro**	0 ± 1	2 ± 1		
		**Neck**	75 ± 9	95 ± 4		
		**Tail**	93 ± 6	16 ± 5		
	**C**	**Acrosome**	12 ± 2 ***	15 ± 2 ***	125 ± 7 ***	123 ± 5 ***
		**Sub-acro**	7 ± 2 ***	6 ± 2 *		
		**Neck**	89 ± 10 **	90 ± 6		
		**Tail**	93 ± 4	12 ± 5		
	**Asta**	**Acrosome**	28 ± 7 ^††^	22 ± 1 ^††^	141 ± 4 ^††^	139 ± 6 ^††^
		**Sub-acro**	32 ± 9 ^††^	21 ± 4 ^††^		
		**Neck**	92 ± 7	96 ± 3		
		**Tail**	90 ± 6	13 ± 4		
	**PP2**	**Acrosome**	9 ± 1 ^†^	8 ± 2 ^††^	2 ± 1 ^‡^	1 ± 2 ^‡^
		**Sub-acro**	5 ± 2	1 ± 1 ^†^		
		**Neck**	71 ± 9 ^†^	68 ± 11 ^††^		
		**Tail**	84 ± 8 ^†^	9 ± 4		
	**Asta + PP2**	**Acrosome**	6 ± 4 ^†^	9 ± 1 ^††^	3 ± 1 ^‡^	1 ± 1 ^‡^
		**Sub-acro**	7 ± 3	3 ± 2		
		**Neck**	82 ± 8	73 ± 7 ^†^		
		**Tail**	83 ± 6 ^†^	9 ± 3		

These data strengthen the efficacy of Asta in ameliorating sperm unable to undergo correct capacitation leading to AR. On the other hand, normal sperm did not benefit from Asta positive effects for AR, probably due to the fact that sperm cells were working to the best of their capacity so that any further improvement was quite impossible. In any case, in both groups the presence of Asta in the incubation medium decreased the number of NVC (Table 1), thus confirming the efficacy of this compound to prevent sperm apoptosis.

PP2, an inhibitor of the Src family kinase, induced cell denaturation leading to apoptosis. This confirmed the involvement of one or more members of this family, not only in the capacitating process but also in spermatozoa survival. Previous investigations [32,33] indicated p60src as responsible for capacitation progress in human sperm, though this enzyme was found predominantly in the neck, tail and pre-acrosome region. In the present study, Lyn, rather than p60src, better represents the key enzyme to the Tyr-P process involving sperm head. In fact, Lyn migrates from neck to the acrosome region, probably enveloped in raft caveolae/microdomains. Only when located in acrosome, Lyn activity highly increased, thus allowing the Tyr-P of the head.

**Figure 3:**
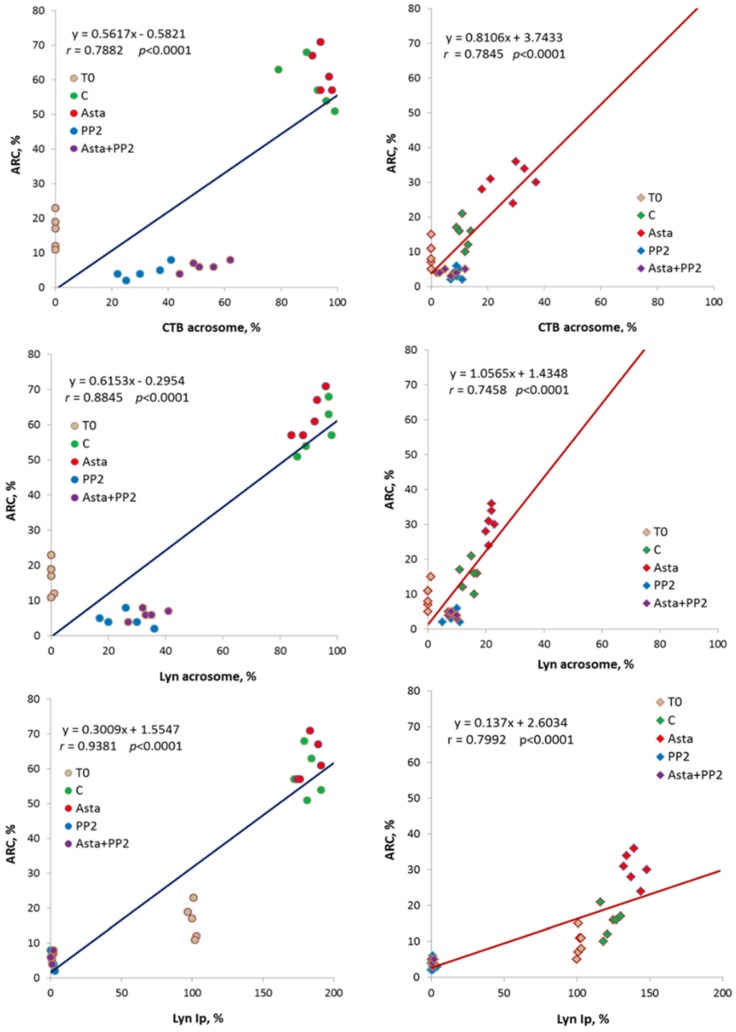
Correlations between acrosomal CTB, acrosomal Lyn and Lyn-Ip activity (Lyn-Ip %) values *vs.* ARC in both groups (CG on left, PG on right). Linear regression parameters are indicated, as well as Pearson’s coefficient of correlation *r* (*p* < 0.0001 in all cases).

Statistical analysis indicated close relationships between acrosomal CTB, acrosomal Lyn and Lyn-Ip activity values *vs.* ARC, as showed by the statistically significant linear regressions obtained through data points (*p* < 0.0001) in both groups (Figure 3). Moreover, the trend lines show clearly the phenomenon of increase in all the considered parameters following Asta treatment in PG.

Previous studies have demonstrated that AR closely depends on a controlled range of ROS generation leading to the Tyr-P process of the sperm head [18,19]. In sperm unable to produce them or in the presence of strong anti-oxidants, such as ascorbic acid, spermatozoa undergo a kind of general impairment, which prevents not only their ability to undergo AR but also cell survival [18]. This close relation between the endogenous ROS content and sperm viability were further confirmed in the present study. In fact, when CG sperm was incubated in the presence of AA capacitation was prevented as shown by the inhibition of ROS production (Supplementary Figure S1, panel A), Tyr-P and AR (Supplementary Table S2). On the other hand, when PG sperm was treated in the presence of H_2_O_2_ (0.25 μM) to ameliorate ROS content (Supplementary Figure S1, panel B) and trigger capacitation, no improvement either in Tyr-P level and distribution or ARC was shown (Supplementary Table S2). In this case, it is hypothesized that the impairment present in PG sperm, being inserted in the membrane, was not achievable by exogenously added ROS.

In the present study, sperm samples were incubated in a buffer which guaranteed an optimal ROS production in normal CG as shown by the high ARC percentage but not sufficient to trigger capacitation in PG. In any case, low percentages of apoptosis (NVC) were ensured, and, more interestingly, viability impairment was prevented, as indicated by the net increase of ARC following Asta addition, thus indicating that cells were in a sort of resting conditions ready for a potential successive activation. This important aspect shed light also to the possible mechanism of action of Asta. In fact, the main difference evidenced in PG was the failure in raft shifting and relocation, compared to the high percentage of CG cells easily achieving capacitating membrane adjustment. By displacing rafts together with rafts-inserted-Lyn to the acrosome region, Asta markedly induced the conformational change required for AR, thus bypassing the impairment present in these cells for the capacitation-related arrangement. Possibly Asta, by inserting in the lipid bilayer, disengages rafts from the blocks that maintain the membrane in non-capacitated form, thus allowing both rafts and relative inserted proteins to reach the capacitated-related relocation. It is hypothesized that mechanisms other than cholesterol extraction may be involved in the membrane reorganization induced by capacitation, since Asta inserts itself into the bilayer, probably disrupting protein-protein or protein-lipid interactions, thus allowing/facilitating both the correct engagement of proteins in newly formed complexes, such as in the case of actin polymerization [34], and the correct relocation of enzymes, such as Lyn, to the acrosome region. This Lyn relocation had to be accompanied by its activation in order to achieve correct level of Tyr-P. Only when Tyr-P process involved the acrosome region capacitation was accomplished, as demonstrated by the high percentage of ARC in the corresponding cells. Taken together, these results show for the first time the close relation between endogenous ROS correct generation and raft migration to the sperm head as “*sine qua non condicio*” for the successive AR. In addition, Lyn, probably embedded and co-migrating with lipid rafts, is activated and catalyzes Tyr-P of proteins localized in the sperm head, third and further step of sperm preparation to achieve AR. When ROS content is insufficient [13,18] (PG), rafts cannot migrate, thus blocking successive phases, and this may be the limiting step for distinguishing PG from CG samples, even if routine test values fall into the normal range.

## 3. Experimental Section

### 3.1. Semen Collection and Analysis

Twenty-four healthy male donors of proven fertility (with 1–3 children) (age range: 24–37 years, average age: 31.4 years) together with patients (*n* = 27) (age range, 26–38 years; average age: 32.5 years), from couples who had failed to conceive after at least one year of regular unprotected intercourse, were enrolled at the Department of Medicine-Endocrinology, University of Padova, Italy. The female partners of the patients resulted normal after gynaecologic evaluations. After three days of abstinence, semen samples were collected by masturbation in a sterile container and then assessed for sperm parameters. All sperm samples used in this study were normal in terms of sperm count, motility, morphology, volume, fructose level and pH, according to the World Health Organization criteria [35]. All samples presenting any kind of contamination were discarded. This study was approved by the Ethics Committee for Research and Clinical Trials of our University (02-13-2012), and all recruited donors gave their informed written consent and provided detailed lifestyle histories.

### 3.2. Chemicals

Anti-P-Tyr mouse monoclonal, goat anti-mouse and anti-rabbit IgG-fluorochrome fluorescein isothiocyanate (FITC) conjugate antibodies were purchased from Upstate (Becton Dickinson Italia SpA, Milan, Italy) and Santa Cruz Biotechnology (Heidelberg, Germany), respectively. Anti-Lyn rabbit polyclonal antibody was obtained by Millipore (Temecula, CA, USA). Density gradient (Pure Sperm 40/80) and pure sperm wash buffer (PSW) were obtained from Nidacon International AB (Göteborg, Sweden). Asta was supplied by FERpharma s.r.l. (Milan, Italy). In addition, 12-myristate-13-acetate phorbol ester (PMA) was purchased from Calbiochem (Nottingham, UK) and all other reagents and anti-tubulin antibodies from Sigma-Aldrich (Milan, Italy).

### 3.3. Sample Preparation

After semen analysis, samples were laid on a discontinuous gradient (Pure Sperm 40/80%) and centrifuged at 500× *g* for 30 min at room temperature. The seminal plasma and sperm from the 40% gradient interface were discarded, and the sperm cells from the bottom pellet (80% gradient) were gathered. After gradient separation, sperm samples of each group were collected three by three in a single pool to obtain a sufficient number of cells to perform all tests (ROS, AR status and viability, Tyr-P, *etc.*) for each experiment. Cells were washed with PSW, re-analysed for concentration, motility, viability and morphology, and collected in a single vial (stock sample) at a concentration adjusted to 80 × 10^6^ sperm cells/mL in PSW. Stock samples were prepared in aliquots and analysed immediately (T_0_) or incubated for up to 180 min in capacitating conditions, in the absence (C) or presence of Asta 2 μM from stock solutions of 100 mM dissolved in dimethyl sulphoxide (DMSO) (Asta), PP2 6 μM (PP2), or together (Asta + PP2).

### 3.4. ROS Enhanced Chemiluminescence (ECL)

Production of ROS was measured by the chemiluminescence assay method with luminol (5-amino-2,3-dihydro-1,4-phthalazinedione) as probe [19,36]. Briefly, 2 μL of 25 mM luminol and 4 μL of 10 mg/mL horseradish peroxidase, both prepared in DMSO, were added to 200 μL of a sperm suspension at a concentration of 10 × 10^6^ cells/mL. ROS levels were determined by a luminometer (Fluoroskan Ascent FL, Labsystems, Helsinki, Finland) in the integrated mode for 180 min at 37 °C. Results are expressed as Relative Luminescence Units (RLU) per 2 × 10^6^ sperm cells. Lastly, 2 μL of a 10 mM N-formylmethionyl-leucyl-phenylalanine (FMLP) stock was added and, after a further 10 min of incubation, 4 μL of a 1 nM stock solution of PMA was added, to exclude leukocyte contamination [37]. Only samples with negative response to FLMP and PMA were processed.

### 3.5. Computer Assisted Sperm Analysis (CASA)

Sperm motility and hyperactivation were analysed using a computer-assisted sperm analyzer (CASA). For each sample, the following parameters were evaluated: the percentage of motile spermatozoa and VCL (curvilinear velocity), VAP (average path velocity), VSL (straight-line velocity) and ALH (amplitude of lateral head displacement) to determine the percentage of hyper-activated (HA) cells. Only cells with VCL ≥ 150 μm/s, LIN (VSL/VCL) ≤ 50%, and ALH ≥ 7 μm [36] were considered HA. All measurements were performed at 37 °C. A minimum of 100 cells and 5 fields were analysed for each aliquot [38].

### 3.6. Anti-P-Tyr and Anti-Lyn Evaluations at Confocal Microscopy

Aliquots of sperm (15 × 10^6^ cells) from each sample were washed with phosphate buffer saline (PBS) containing vanadate 1 mM and protease inhibitor cocktail, fixed with 2% (w/v) paraformaldehyde and incubated overnight at 4 °C on slides pre-coated with poly-L-lysine [19]. Slides were rinsed twice with PBS and sperm cells were permeabilized with 0.2% (v/v) Triton X-100 for 15 min at 4 °C and then incubated with anti-P-Tyr or anti-Lyn antibodies for 1 h at 37 °C in a humid chamber. Slides were washed with PBS, stained with anti-mouse or anti-rabbit IgG-FITC conjugate for 1 h at 37 °C in a humid chamber and then rinsed with PBS and mounted. Staining without primary antibody was used as negative control. Fluorescence was detected with the UltraView LCI confocal system (Perkin Elmer, Waltham, MA, USA) equipped with a fluorescence filter set for excitation at 488 nm.

### 3.7. Evaluation of Acrosome Reaction

Acrosome status was monitored with acrosome-specific FITC-labeled peanut (*Arachis hypogaea*) agglutinin (FITC-PNA) in conjunction with DNA-specific fluorochrome propidium iodide (PI) as a viability test [39]. Briefly, in order to induce AR, aliquots (15 × 10^6^ cells) of each sample were incubated for 30 min at 37 °C, in the presence of 10 μM Ca^2+^ ionophore A23187 [19]. Samples contained DMSO, but not ionophore, were used as control. After incubation sperm cells were centrifuged, resuspended in PBS and treated for 10 min at room temperature with 12 μM PI. Sperm was washed with PBS, fixed with 2% (w/v) paraformaldehyde and incubated overnight at 4 °C on poly-l-lysine-treated slides. Permeabilized sperm cells, as described above, were stained with 1 mg FITC-PNA/mL for 15 min at 37 °C in the dark, washed and mounted. At least 200 cells were evaluated for each sample, and fluorescence was detected as described above. Only sperm cells showing evenly distributed fluorescence over the acrosomal region were considered acrosome-intact.

### 3.8. Evaluation of Membrane Rafts

GM1 membrane raft marker was visualized in live human spermatozoa by staining with the cholera toxin subunit B (CTB)-FITC [13,14]. For this purpose, suspensions of cells (15 × 10^6^ cells) from each sample were mixed with an equal volume of CTB (50 μg/mL) and incubated for 15 min at 37 °C. The sperm cells were then washed twice in PBS before being fixed in 2% paraformaldehyde for 30 min, mounted on poly-l-lysine coated glass microscope slides and viewed using the confocal microscope as described above. For each treatment, at least 200 cells were counted and categorized into four different fluorescent patterns (acrosome, sub-acrosome region, neck and tail).

### 3.9. Protein Tyrosine Kinase (PTK) Activity Assays and Distribution

#### 3.9.1. Total Cells Lysate

Sperm cells (20 × 10^6^) from each sample, were resuspended in 300 μL of PBS (containing 1 mM sodium orthovanadate and protease inhibitor cocktail) and treated with 2 mM (final concentration) of 3-[(3-cholamidopropyl)dimethylammonio]-1-propanesulphonate hydrate (CHAPS) [19] at 0 °C for 10 min.

Thirty μL of this total cell lysate (corresponding to about 2 × 10^6^ cells) was assayed PTK activity.

#### 3.9.2. Anti-Lyn Immunoprecipitations (Ip-Lyn)

The remaining volume of total cell lysates were extracted in Igepal buffer (Igepal 1% final concentration, Tris-HCl 20 mM, EDTA 2 mM, NaCl 300 mM EGTA 10 mM, 1 mM sodium orthovanadate and protease inhibitor cocktail) at 4 °C for 1 h in agitation. After centrifugation, supernatants were pre-cleared with protein A-Sepharose, and then incubated overnight at 4 °C with anti-Lyn antibody bound to protein A-Sepharose. Immune complexes were washed three times in 50 mM Tris-HCl pH 7.5, 1 mM vanadate and protease inhibitor cocktail, and subjected to Tyr-protein kinase activity assay.

Tyr-phosphorylation assays were performed by incubating total sperm lysate (2 × 10^6^) or Lyn-Ip for 10 min at 30 °C in 40-μL reaction mixtures containing 50 mM Tris-HCl, pH 7.5, 10 mM MnCl_2_, 20 μM [γ-^33^P]ATP (3 × 10^6^ cpm/nmol) and 0.1 mM vanadate and 200 μM cdc2 peptide, which served as specific substrates for Src family tyrosine kinases. Reactions were stopped by the addition of 2% sodium dodecyl sulphate (SDS) and 1% β-mercaptoethanol (final concentrations) incubated for 5 min at 100 °C, and analyzed by SDS-PAGE and revealed by a Cyclone Storage Phosphor Imager (Perkin Elmer, Downers Grove, IL, USA) [40].

#### 3.9.3. Lyn Distribution

To determine whether Lyn was present in the plasma membrane, head or flagellum, intact spermatozoa (3 × 10^7^ cells) were sonicated three times (30 s followed by a 30 s rest period each) on ice, and heads and flagellar fragments were then separated by a 15 min centrifugation (700× *g*) at 4 °C through a 75% Percoll layer in PSW. Flagellar fragments were recovered at the surface of the Percoll layer while the heads were found in the pellet. The purity of each fraction was assessed by microscopy prior to proceeding to analysis. The supernatant was centrifuged for 10 min (10, 000× *g*, 4 °C) and the resulting supernatant was further centrifuged (100, 000× *g*) to separate the membrane from the cytosol [22]. Each resulting fraction (membranes M, cytosol C, head H and flagella F) was diluted with PBS to the initial volume except for membranes which were concentrated three times, and further processed as described above for total cell lysate. The presence of Lyn was investigated by Western blotting and immuno-revealed with anti-Lyn antibody.

### 3.10. Statistical Analysis

Results are expressed as means ± SD. Comparisons were obtained with Student’s *t-*test for paired or unpaired data and statistical significance was set at *p* < 0.05 (two-tailed). Relationships between pairs of variables were tested by least-squares linear regression. Pearson’s correlation coefficient *r* was used to quantify the strength of the relationships. The statistical significance of *r* was determined by ANOVA. All statistical analyses were performed with JMP^®^ 10 software (SAS Institute, Cary, NC, USA).

## 4. Conclusions

Many efforts have been recently spent in the study of diagnosis and prognosis of mammal infertility, including Tyr-P regulation of capacitating process [41].

In this study, the close relationship among ROS, Tyr-P of sperm head and AR [18,19] was further confirmed, but, more interestingly, our results suggest an interdependence between ROS generation and lipid rafts relocation leading to the acquisition of a correct membrane arrangement for the successive AR. The evidence that only samples generating the correct amount of ROS and presenting rafts concentrated mainly on the head are able to achieve AR suggests that ROS may be responsible for lipid rearrangement. ROS, in fact, would induce lipid peroxidation, thus disrupting lipid-lipid interaction and allowing rafts to shift over the head where they concentrate.

Hereby we show that Asta induced raft migration even in the absence of the correct ROS generation (PG), bypassing the ROS-induced membrane alterations. It is likely that inserting Asta within the bilayer enables mechanical translocation of rafts, setting them free from the blocking sites.

However, raft shifting is only a part of the mechanism involved in this membrane remodeling, the second being represented by Lyn co-migration with rafts. This Src family kinase, in fact, has the main role in the sperm head Tyr-P, another condition for the AR to occur [18,19]. In this study, Lyn location and activation were demonstrated to be prerequisite for the successive acrosome reaction.

## Supplementary Files

Supplementary File 1
