# Supplementary material for: Astaxanthin Improves Human Sperm Capacitation by Inducing Lyn Displacement and Activation
Source: Mar Drugs. 2015 Aug 25;13(9):5533–51. doi: 10.3390/md13095533 (PMC4584338; doi:10.3390/md13095533)
Supplement: Supplementary File 1 [file marinedrugs-13-05533-s001.doc]

**Supplementary Materials**

**
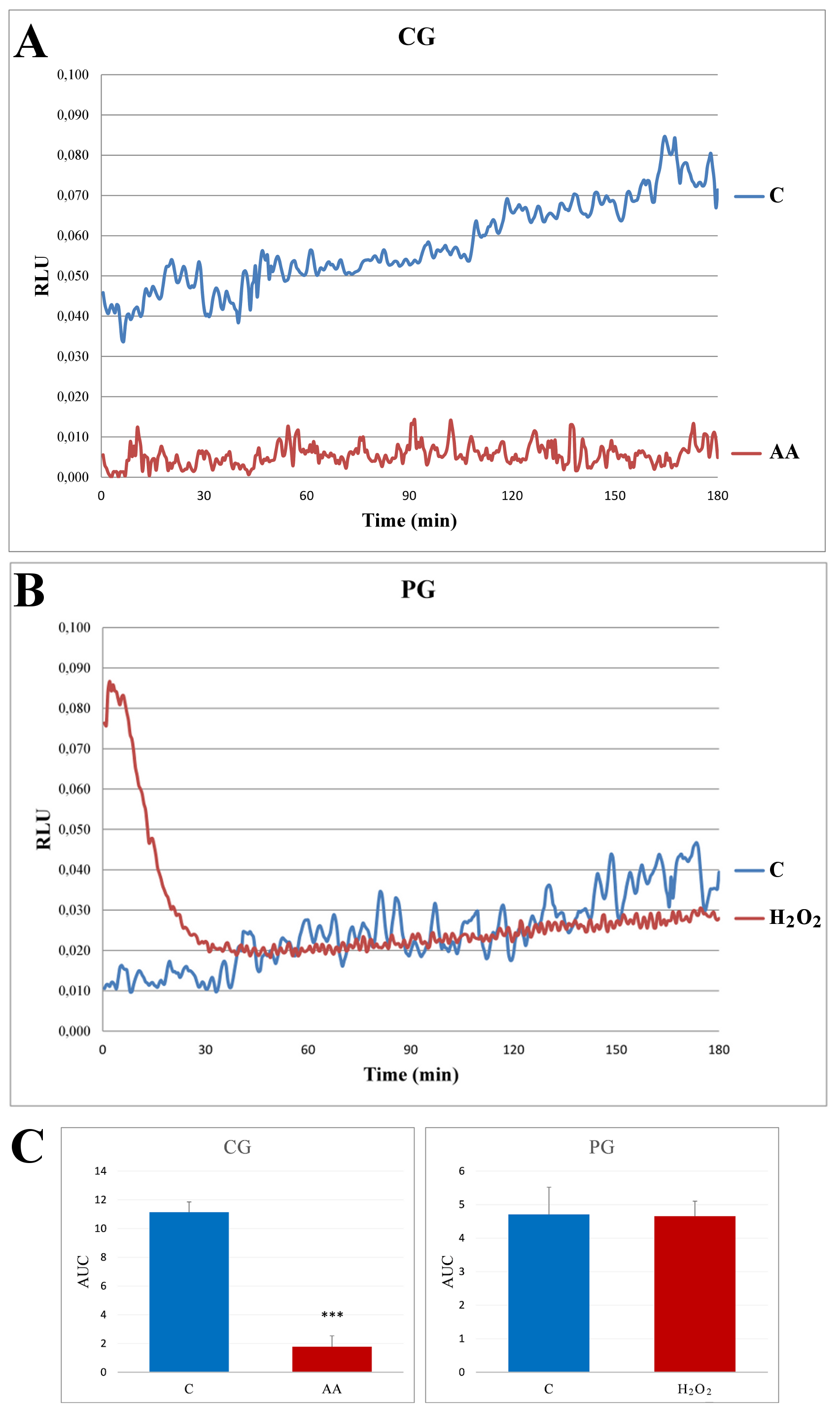
**

**Figure S1.** ROS generation curves of sperm samples and effects of Ascorbic Acid (AA) or H_2_O_2_. Sperm from 3 volunteers for each experiment was collected to form a pool with a sufficient number of cells. Sperm was incubated for up to 180 min in capacitating conditions in the absence (C) or presence of AA (1 mM) for cells from CG,
or H_2_O_2_ (0.6 M) for PG . Luminol chemiluminescence was monitored during sperm capacitation. Results are expressed as moving averages of Relative Luminescence Units (RLU)/30 s for 2 × 10^6^ cells. The figure is representative of 3 separate experiments, conducted on samples belonging to the control group (CG, panel **A**) or the patient group (PG, panel **B**). Detection was carried out in triplicate. Graphs in panel **C** show AUC of the RLU data in both groups. *** *p* < 0.001 *vs.* C; Student’s *t* test.


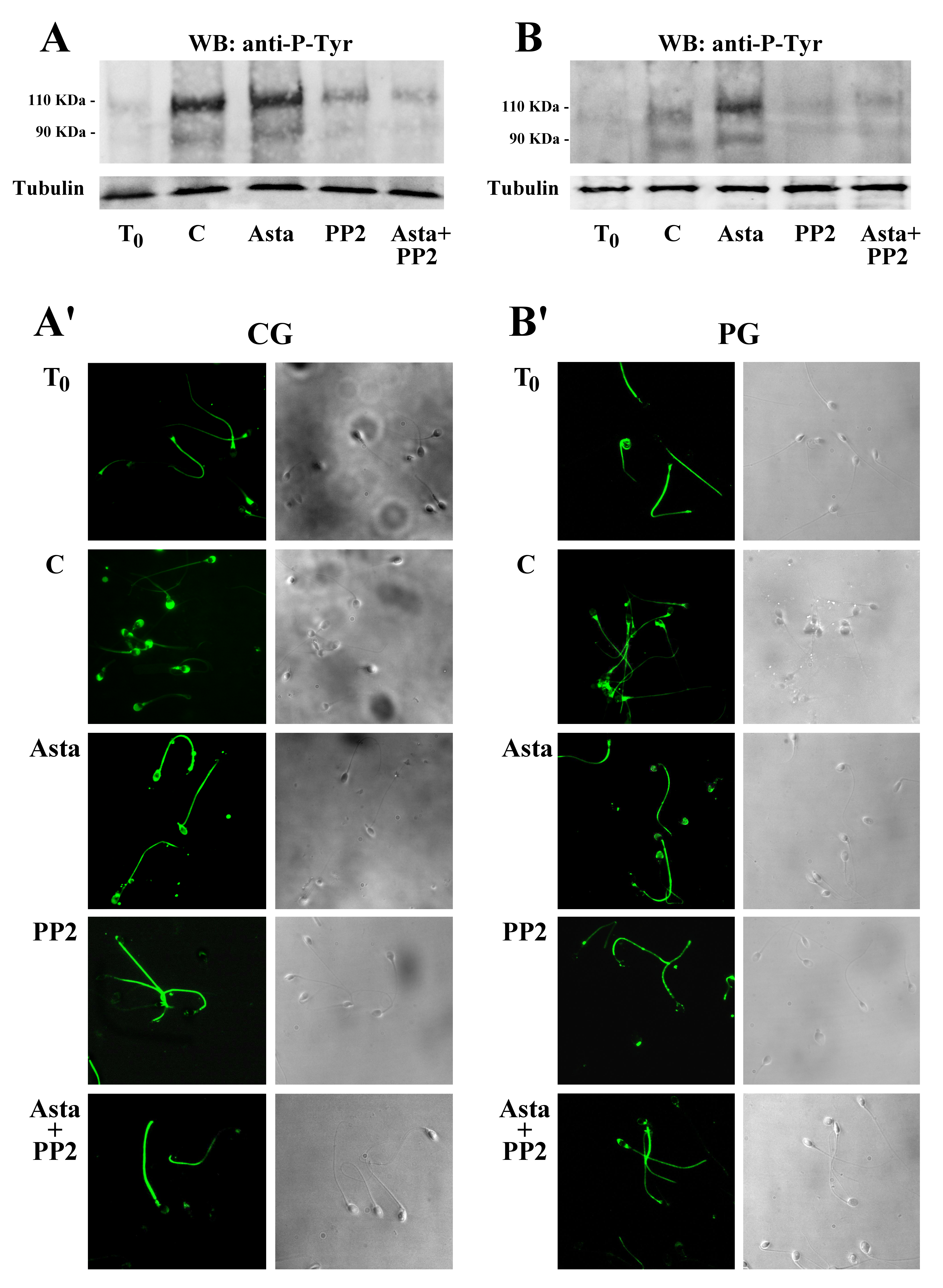


**Figure S2.** Panels **A** and **B**: Western blotting analysis of Tyr-P proteins in total cell lysates from CG (panel **A**) or PG (panel **B**) sperm was carried out in samples T_0_, C, Asta, PP2 and Asta + PP2. Cells (1 × 10^6^) incubated in the different conditions were treated with 2 mm (final concentration) of 3-[(3-cholamidopropyl) dimethylammonio]-1-propanesulphonate hydrate (CHAPS), solubilized by adding sodium dodecyl sulphate (SDS) and
β-mercaptoethanol (2% final concentration), boiled at 100 °C for 5 min and subjected to SDS-PAGE (10%). Proteins were electro-transferred to a nitrocellulose membrane and immuno-revealed with anti-P-Tyr antibody, or anti-Tubulin as loading control. Panels **A’** and **B’**: sperm from control group (CG, panel **A’**) and patient group (PG, panel **B’**),
at T_0_ or incubated in capacitating conditions for 180 min in absence (C) or presence
of Asta (2 M), PP2 (6 M) or Asta + PP2, were analysed for anti-Tyr-P labelling
by immunofluorescence cytochemistry as described in Methods. Fluorescent and corresponding phase-contrast images for each conditions are shown. The figure is representative of 11 separate experiments conducted in triplicate.

**Table S1.** Sperm motility and hyperactivation were analysed using a computer-assisted sperm analyzer (CASA). For each sample, the following parameters were evaluated: the percentage of motile spermatozoa and VCL (curvilinear velocity), VAP (average path velocity), VSL (straight-line velocity) and ALH (amplitude of lateral head displacement) to determine the percentage of hyper-activated (HA) cells. Only cells with VCL ≥ 150 µm/s, LIN (VSL/VCL) ≤ 50%, and ALH ≥ 7 µm [36] were considered HA. All measurements were performed at 37 °C. A minimum of 100 cells and 5 fields were analysed for each aliquot. Values are expressed as means ± SD. ^†^ *p* < 0.05 comparison between various samples *vs.* C as reference; Student’s *t*-test for paired data. * *p* < 0.05; ** *p* < 0.01;
*** *p* < 0.001 comparison C *vs.* T_0_; Student’s *t-*test for paired data.

| **Groups** | **Parameter** | **T0** | **C** | **Asta** | **PP2** | **Asta + PP2** |
| --- | --- | --- | --- | --- | --- | --- |
| **C** | **Motility** | 84 ± 4 | 74 ± 5 * | 76 ± 6 | 72 ± 6 | 75 ± 4 |
|  | **HA** | 6 ± 3 | 26 ± 4 *** | 24 ± 5 | 23 ± 3 | 24 ± 3 |
| **PG** | **Motility** | 83 ± 5 | 71 ± 4 ** | 72 ± 3 | 70 ± 3 | 72 ± 5 |
|  | **HA** | 5 ± 2 | 24 ± 4 *** | 27 ± 4 ^†^ | 25 ± 4 | 24 ± 6 |

**Table S2.** Sperm cells from control group (CG) or patient group (PG) at T_0_, or incubated for 180 min in capacitating conditions in the absence (C) in the presence of AA (1 mM) for CG, or H_2_O_2_ (0.25 M) for PG were analysed for Tyr-P pattern, acrosome-reacted cells (ARC) and viability (non-viable cells, NVC) by immunofluorescence cytochemistry (see Methods). Number of cells expressed as % of total number of cells showing Tyr-P in
any part of cell body or in head, were detected and reported as Tyr-P cells and Tyr-P head, respectively. Percentages of cells undergoing acrosome reaction (ARC) or NVC were
also reported. Values are expressed as means ± SD. ^†^ *p* < 0.05 comparison between AA
or H_2_O_2_ *vs.* C as reference; Student’s *t-*test for paired data. ** *p* < 0.01 comparison C *vs.* T_0_; Student’s *t-*test for paired data.

| **Group** | **Parameter** | **T0** | **C** | **AA** | **H2O2** |
| --- | --- | --- | --- | --- | --- |
| **CG** | **Tyr-P cells** | 92 ± 3 | 94 ± 4 | 74 ± 5 ^†^ | **-** |
|  | **Tyr-P head** | 12 ± 3 | 67 ± 6 ** | 34 ± 7 ^†^ | **-** |
|  | **ARC** | 17 ± 4 | 60 ± 6 ** | 27 ± 4 ^†^ | **-** |
|  | **NVC** | 6 ± 2 | 7 ± 3 | 15 ± 3 | **-** |
| **PG** | **Tyr-P cells** | 46 ± 5 | 56 ± 7 | **-** | 51 ± 6 |
|  | **Tyr-P head** | 7 ± 5 | 21 ± 3 | **-** | 9 ± 2 ^†^ |
|  | **ARC** | 9 ± 3 | 16 ± 4 | **-** | 10 ± 2 |
|  | **NVC** | 14 ± 4 | 22 ± 3 ** | **-** | 33 ± 4 |

© 2015 by the authors; licensee MDPI, Basel, Switzerland. This article is an open access article distributed under the terms and conditions of the Creative Commons Attribution license (http://creativecommons.org/licenses/by/4.0/).
